# 4,4′-Bipyridine–2-(carb­oxy­methyl­sulfan­yl)pyridine-3-carb­oxy­lic acid (1/1)

**DOI:** 10.1107/S1600536810048385

**Published:** 2010-11-27

**Authors:** Xian-Rong Jiang, Xiao-Juan Wang, Yun-Long Feng

**Affiliations:** a Zhejiang Key Laboratory for Reactive Chemistry on Solid Surfaces, Institute of Physical Chemistry, Zhejiang Normal University, Jinhua, Zhejiang 321004, People’s Republic of China

## Abstract

In the title co-crystal, C_10_H_8_N_2_·C_8_H_7_NO_4_S, the formate group is coplanar with the pyridyl ring of the acid [dihedral angle = 6.2 (7)°], while the carb­oxy­methyl­sulfanyl group makes a C—S—C—C torsion angle of 70.2 (1)° with the pyridine ring. The dihedral angle between the pyridyl rings of the 4,4′-bipyridine mol­ecule is 27.4 (1)°. The acid and the 4,4′-bipyridine mol­ecules are involved in hydrogen bonding *via* carb­oxy­lic O and pyridyl N atoms. The structure is further consolidated by inter­molecular C—H⋯O hydrogen bonds, generating a three-dimensional network.

## Related literature

For related structures, see: Wang & Feng (2010 ▶); Zhu *et al.* (2002 ▶); Smith & Sagatys (2003 ▶).
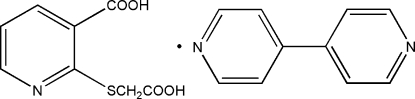


## Experimental

### 

#### Crystal data

C_10_H_8_N_2_·C_8_H_7_NO_4_S
*M* *_r_* = 369.40
Monoclinic, 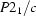

*a* = 9.3684 (3) Å
*b* = 10.3044 (3) Å
*c* = 18.2264 (5) Å
β = 106.494 (2)°
*V* = 1687.09 (9) Å^3^
*Z* = 4
Mo *K*α radiation
μ = 0.22 mm^−1^
*T* = 296 K
0.41 × 0.25 × 0.10 mm

#### Data collection

Bruker APEXII area-detector diffractometer
Absorption correction: multi-scan (*SADABS*; Sheldrick, 1996 ▶) *T*_min_ = 0.935, *T*_max_ = 0.978
24834 measured reflections
3927 independent reflections
3106 reflections with *I* > 2σ(*I*)
*R*_int_ = 0.028

#### Refinement

*R*[*F* ^2^ > 2σ(*F* ^2^)] = 0.038
*wR*(*F* ^2^) = 0.144
*S* = 1.08
3927 reflections
241 parameters
2 restraints
H atoms treated by a mixture of independent and constrained refinement
Δρ_max_ = 0.27 e Å^−3^
Δρ_min_ = −0.25 e Å^−3^

### 

Data collection: *APEX2* (Bruker, 2006 ▶); cell refinement: *SAINT* (Bruker, 2006 ▶); data reduction: *SAINT*; program(s) used to solve structure: *SHELXS97* (Sheldrick, 2008 ▶); program(s) used to refine structure: *SHELXL97* (Sheldrick, 2008 ▶); molecular graphics: *SHELXTL* (Sheldrick, 2008 ▶); software used to prepare material for publication: *SHELXTL*.

## Supplementary Material

Crystal structure: contains datablocks I, global. DOI: 10.1107/S1600536810048385/pv2357sup1.cif

Structure factors: contains datablocks I. DOI: 10.1107/S1600536810048385/pv2357Isup2.hkl

Additional supplementary materials:  crystallographic information; 3D view; checkCIF report

## Figures and Tables

**Table 1:** Hydrogen-bond geometry (Å, °)

*D*—H⋯*A*	*D*—H	H⋯*A*	*D*⋯*A*	*D*—H⋯*A*
O1—H1*B*⋯N2^i^	0.86 (2)	1.79 (2)	2.6564 (18)	178 (2)
O3—H3*B*⋯N3^ii^	0.86 (2)	1.82 (2)	2.6618 (18)	167 (2)
C4—H4*A*⋯O4^iii^	0.93	2.55	3.213 (2)	128
C15—H15*A*⋯O2^ii^	0.93	2.39	3.0664 (19)	130
C18—H18*A*⋯o2^ii^	0.93	2.70	3.232 (2)	117

Symmetry codes: (i) 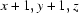; (ii) 
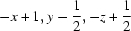
; (iii) 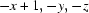.

**Fig. 1.**
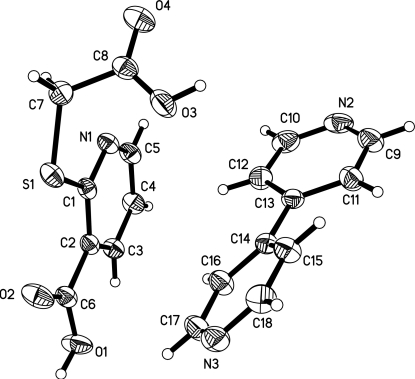
Perspective view of the structure of (I), showing the atom-numbering scheme. Displacement ellipsoids are drawn at the 30% probability level.

**Fig. 2.**
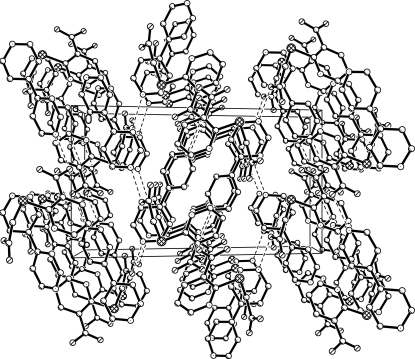
A unit cell packing of (I); intermolecular hydrogen bonds have been depicted by dashed lines.

## supplementary crystallographic information

### Comment

The crystal structures of a number of mercaptonicotinic derivatives have been reported, such as 2-(carboxymethylsulfanyl)pyridine-3-carboxylic acid monohydrate (Wang *et al.*, 2010), bis(3-carboxypyrid-2-yl)disulfide monohydrate (Zhu *et al.*, 2002) and ammonium 2-mercaptopyridine-3-carboxylate monohydrate (Smith *et al.*, 2003). In an attempt to synthesize a cobalt complex with 2-(carboxymethylsulfanyl)pyridine-3-carboxylic acid and 4,4'-bipyridine, we obtained the title compound, (I), unexpectedly. In this article, we report the crystal structure of (I).

The title compound is composed of 2-(carboxymethylsulfanyl)pyridine-3-carboxylic acid (C_8_H_7_NO_4_S) and 4,4'-bipyridine (C_10_H_8_N_2_) (Fig. 1). In the acid moiety, the formate group is coplanar with the pyridyl ring, while the carboxymethylsulfanyl group is almost vartical to the plane formed by the pyridine ring atoms with torsion angle, C1—S1—C7—C8, 70.2 (1)°. The dihedral angle between the pyridyl rings of the 4,4'-bipyridine molecule is 27.4 (1)°. The acid and the 4,4'-bipyridine molecules are involved in hydrogen bonding *via* carboxylic O and pyridyl N atoms. The structure is further consolidated by intermolecular hydrogen bonds of type C—H···O (Fig. 2 and Tab. 1).

### Experimental

2-(Carboxymethylsulfanyl)pyridine-3-carboxylic acid was prepared according to the literature method (Wang *et al.*, 2010). A mixture of CoCl_2_.6H_2_O (0.2379 g, 1.0 mmol), 4,4'-bipyridine (0.0468 g, 0.3 mmol) and 2-(carboxymethylsulfanyl)pyridine-3-carboxylic acid (0.2134 g, 1.0 mmol) was dissolved in 10.0 ml of distilled water and 3.0 ml ethanol at 328 K. The resulting solution was stirred and refluxed under basic condition for 2 h, the mixture was cooled to room temperature and filtered. Single crystals suitable for X-ray diffraction were obtained from the mother liquor by slow evaporation at room temperature for several days.

### Refinement

The carbon-bound H-atoms were positioned geometrically and included in the refinement using a riding model with C—H = 0.93 and 0.97 Å for aryl and methylene H-atoms and *U*_iso_(H) = 1.2*U*_eq_(C). The oxygen-bound H-atoms was located in a difference Fourier map and refined with the O—H distance restrained to 0.85 (2) Å and *U*_iso_(H) = 1.2*U*_eq_(O).

### Crystal data

**Table:**

C_10_H_8_N_2_·C_8_H_7_NO_4_S	*F*(000) = 768
*M**_r_* = 369.40	*D*_x_ = 1.454 Mg m^−^^3^
Monoclinic, *P*2_1_/*c*	Mo *K*α radiation, λ = 0.71073 Å
Hall symbol: -P 2ybc	Cell parameters from 7490 reflections
*a* = 9.3684 (3) Å	θ = 2.3–27.7°
*b* = 10.3044 (3) Å	µ = 0.22 mm^−^^1^
*c* = 18.2264 (5) Å	*T* = 296 K
β = 106.494 (2)°	Block, colourless
*V* = 1687.09 (9) Å^3^	0.41 × 0.25 × 0.10 mm
*Z* = 4	

### Data collection

**Table:**

Bruker APEXII area-detector diffractometer	3927 independent reflections
Radiation source: fine-focus sealed tube	3106 reflections with *I* > 2σ(*I*)
graphite	*R*_int_ = 0.028
ω scans	θ_max_ = 27.7°, θ_min_ = 2.3°
Absorption correction: multi-scan (*SADABS*; Sheldrick, 1996)	*h* = −12→12
*T*_min_ = 0.935, *T*_max_ = 0.978	*k* = −13→13
24834 measured reflections	*l* = −23→23

### Refinement

**Table:**

Refinement on *F*^2^	Primary atom site location: structure-invariant direct methods
Least-squares matrix: full	Secondary atom site location: difference Fourier map
*R*[*F*^2^ > 2σ(*F*^2^)] = 0.038	Hydrogen site location: inferred from neighbouring sites
*wR*(*F*^2^) = 0.144	H atoms treated by a mixture of independent and constrained refinement
*S* = 1.08	*w* = 1/[σ^2^(*F*_o_^2^) + (0.1*P*)^2^] where *P* = (*F*_o_^2^ + 2*F*_c_^2^)/3
3927 reflections	(Δ/σ)_max_ < 0.001
241 parameters	Δρ_max_ = 0.27 e Å^−^^3^
2 restraints	Δρ_min_ = −0.25 e Å^−^^3^

### Special details

**Table:**

Geometry. All e.s.d.'s (except the e.s.d. in the dihedral angle between two l.s. planes) are estimated using the full covariance matrix. The cell e.s.d.'s are taken into account individually in the estimation of e.s.d.'s in distances, angles and torsion angles; correlations between e.s.d.'s in cell parameters are only used when they are defined by crystal symmetry. An approximate (isotropic) treatment of cell e.s.d.'s is used for estimating e.s.d.'s involving l.s. planes.
Refinement. Refinement of *F*^2^ against ALL reflections. The weighted *R*-factor *wR* and goodness of fit *S* are based on *F*^2^, conventional *R*-factors *R* are based on *F*, with *F* set to zero for negative *F*^2^. The threshold expression of *F*^2^ > σ(*F*^2^) is used only for calculating *R*-factors(gt) *etc*. and is not relevant to the choice of reflections for refinement. *R*-factors based on *F*^2^ are statistically about twice as large as those based on *F*, and *R*- factors based on ALL data will be even larger.

### Fractional atomic coordinates and isotropic or equivalent isotropic displacement parameters (Å^2^)

**Table:**

	*x*	*y*	*z*	*U*_iso_*/*U*_eq_	
S1	0.58161 (5)	0.38154 (4)	0.14705 (2)	0.04516 (16)	
O1	0.80222 (16)	0.58013 (13)	−0.00541 (8)	0.0686 (4)	
H1B	0.837 (2)	0.6539 (17)	0.0143 (12)	0.082*	
O2	0.72352 (16)	0.57771 (12)	0.09792 (7)	0.0640 (4)	
O3	0.70285 (16)	0.13228 (12)	0.22294 (9)	0.0705 (4)	
H3B	0.740 (2)	0.0553 (17)	0.2308 (13)	0.085*	
O4	0.49171 (15)	0.02161 (13)	0.18621 (8)	0.0722 (4)	
N1	0.52552 (13)	0.21214 (12)	0.03258 (7)	0.0418 (3)	
C1	0.58945 (15)	0.32661 (14)	0.05633 (7)	0.0368 (3)	
C2	0.65886 (15)	0.40173 (13)	0.01174 (8)	0.0382 (3)	
C3	0.65803 (17)	0.35450 (15)	−0.05940 (9)	0.0439 (4)	
H3A	0.7020	0.4022	−0.0905	0.053*	
C4	0.59198 (17)	0.23668 (16)	−0.08426 (8)	0.0472 (4)	
H4A	0.5906	0.2035	−0.1319	0.057*	
C5	0.52829 (17)	0.17006 (15)	−0.03640 (9)	0.0453 (4)	
H5A	0.4842	0.0904	−0.0530	0.054*	
C6	0.73076 (16)	0.52758 (14)	0.03952 (8)	0.0424 (3)	
C7	0.48104 (17)	0.25100 (16)	0.17466 (8)	0.0467 (4)	
H7A	0.4508	0.2794	0.2187	0.056*	
H7B	0.3909	0.2367	0.1333	0.056*	
C8	0.5588 (2)	0.12241 (15)	0.19413 (9)	0.0475 (4)	
N2	−0.08632 (19)	−0.19297 (15)	0.05293 (10)	0.0635 (4)	
N3	0.16287 (17)	0.40694 (14)	0.23065 (9)	0.0560 (4)	
C9	0.0353 (2)	−0.18397 (18)	0.11218 (13)	0.0652 (5)	
H9A	0.0884	−0.2595	0.1296	0.078*	
C10	−0.1626 (2)	−0.0845 (2)	0.03031 (12)	0.0622 (5)	
H10A	−0.2482	−0.0888	−0.0108	0.075*	
C11	0.08680 (19)	−0.07012 (17)	0.14907 (10)	0.0551 (4)	
H11A	0.1739	−0.0690	0.1893	0.066*	
C12	−0.12119 (17)	0.03452 (18)	0.06467 (9)	0.0529 (4)	
H12A	−0.1787	0.1079	0.0472	0.064*	
C13	0.00740 (16)	0.04348 (15)	0.12563 (9)	0.0415 (3)	
C14	0.05830 (16)	0.16981 (15)	0.16269 (8)	0.0401 (3)	
C15	0.14430 (19)	0.17679 (16)	0.23814 (9)	0.0500 (4)	
H15A	0.1683	0.1018	0.2675	0.060*	
C16	0.02397 (18)	0.28557 (16)	0.12271 (10)	0.0513 (4)	
H16A	−0.0352	0.2859	0.0722	0.062*	
C17	0.0788 (2)	0.39998 (17)	0.15887 (11)	0.0584 (5)	
H17A	0.0554	0.4768	0.1313	0.070*	
C18	0.19387 (19)	0.29589 (19)	0.26931 (9)	0.0558 (4)	
H18A	0.2520	0.2990	0.3200	0.067*	

### Atomic displacement parameters (Å^2^)

**Table:**

	*U*^11^	*U*^22^	*U*^33^	*U*^12^	*U*^13^	*U*^23^
S1	0.0619 (3)	0.0338 (2)	0.0371 (2)	0.00022 (15)	0.00974 (18)	−0.00146 (13)
O1	0.0904 (9)	0.0499 (7)	0.0768 (9)	−0.0340 (7)	0.0421 (7)	−0.0215 (7)
O2	0.0957 (9)	0.0453 (7)	0.0505 (7)	−0.0223 (6)	0.0201 (6)	−0.0118 (5)
O3	0.0620 (8)	0.0418 (7)	0.0978 (11)	0.0049 (5)	0.0069 (7)	0.0182 (7)
O4	0.0866 (9)	0.0450 (7)	0.0837 (9)	−0.0169 (6)	0.0219 (7)	−0.0054 (6)
N1	0.0473 (7)	0.0326 (6)	0.0419 (6)	−0.0030 (5)	0.0066 (5)	−0.0008 (5)
C1	0.0382 (7)	0.0311 (7)	0.0360 (7)	0.0029 (5)	0.0021 (5)	0.0002 (5)
C2	0.0377 (7)	0.0323 (7)	0.0402 (7)	0.0007 (5)	0.0038 (6)	−0.0013 (6)
C3	0.0503 (8)	0.0385 (8)	0.0432 (8)	−0.0029 (6)	0.0136 (7)	−0.0021 (6)
C4	0.0578 (9)	0.0420 (9)	0.0402 (7)	−0.0048 (7)	0.0113 (6)	−0.0091 (6)
C5	0.0513 (8)	0.0337 (8)	0.0446 (8)	−0.0048 (6)	0.0035 (6)	−0.0057 (6)
C6	0.0441 (7)	0.0346 (7)	0.0441 (8)	−0.0022 (6)	0.0053 (6)	−0.0012 (6)
C7	0.0519 (8)	0.0475 (9)	0.0423 (7)	0.0013 (7)	0.0159 (6)	0.0000 (7)
C8	0.0648 (10)	0.0396 (9)	0.0400 (8)	−0.0031 (7)	0.0180 (7)	0.0010 (6)
N2	0.0739 (10)	0.0459 (9)	0.0798 (11)	−0.0249 (7)	0.0365 (8)	−0.0189 (8)
N3	0.0591 (8)	0.0457 (8)	0.0636 (9)	−0.0107 (6)	0.0180 (7)	−0.0158 (7)
C9	0.0743 (12)	0.0388 (9)	0.0883 (14)	−0.0044 (8)	0.0326 (11)	−0.0061 (9)
C10	0.0542 (10)	0.0619 (12)	0.0694 (12)	−0.0208 (8)	0.0157 (9)	−0.0180 (10)
C11	0.0557 (10)	0.0412 (9)	0.0656 (11)	−0.0013 (7)	0.0129 (8)	−0.0010 (7)
C12	0.0454 (8)	0.0477 (9)	0.0611 (10)	−0.0055 (7)	0.0077 (7)	−0.0079 (8)
C13	0.0412 (7)	0.0378 (8)	0.0466 (7)	−0.0066 (6)	0.0141 (6)	−0.0018 (6)
C14	0.0396 (7)	0.0361 (8)	0.0445 (7)	−0.0038 (6)	0.0114 (6)	−0.0025 (6)
C15	0.0582 (9)	0.0453 (9)	0.0437 (8)	−0.0038 (7)	0.0097 (7)	0.0010 (7)
C16	0.0546 (9)	0.0411 (9)	0.0513 (8)	−0.0014 (7)	0.0035 (7)	0.0008 (7)
C17	0.0675 (11)	0.0355 (9)	0.0692 (11)	−0.0010 (7)	0.0143 (9)	0.0013 (8)
C18	0.0603 (10)	0.0592 (11)	0.0447 (8)	−0.0069 (8)	0.0098 (7)	−0.0095 (8)

### Geometric parameters (Å, °)

**Table:**

S1—C1	1.7688 (14)	N2—C10	1.328 (3)
S1—C7	1.7943 (17)	N2—C9	1.332 (3)
O1—C6	1.3127 (19)	N3—C17	1.324 (2)
O1—H1B	0.864 (16)	N3—C18	1.332 (2)
O2—C6	1.2023 (19)	C9—C11	1.370 (2)
O3—C8	1.305 (2)	C9—H9A	0.9300
O3—H3B	0.861 (16)	C10—C12	1.382 (2)
O4—C8	1.2015 (19)	C10—H10A	0.9300
N1—C5	1.3373 (19)	C11—C13	1.388 (2)
N1—C1	1.3379 (18)	C11—H11A	0.9300
C1—C2	1.408 (2)	C12—C13	1.391 (2)
C2—C3	1.383 (2)	C12—H12A	0.9300
C2—C6	1.4827 (19)	C13—C14	1.482 (2)
C3—C4	1.379 (2)	C14—C15	1.384 (2)
C3—H3A	0.9300	C14—C16	1.387 (2)
C4—C5	1.372 (2)	C15—C18	1.377 (2)
C4—H4A	0.9300	C15—H15A	0.9300
C5—H5A	0.9300	C16—C17	1.377 (2)
C7—C8	1.505 (2)	C16—H16A	0.9300
C7—H7A	0.9700	C17—H17A	0.9300
C7—H7B	0.9700	C18—H18A	0.9300
			
C1—S1—C7	100.76 (7)	C17—N3—C18	117.10 (15)
C6—O1—H1B	107.7 (15)	N2—C9—C11	123.95 (18)
C8—O3—H3B	108.5 (15)	N2—C9—H9A	118.0
C5—N1—C1	117.62 (13)	C11—C9—H9A	118.0
N1—C1—C2	122.38 (13)	N2—C10—C12	123.33 (17)
N1—C1—S1	116.80 (11)	N2—C10—H10A	118.3
C2—C1—S1	120.81 (11)	C12—C10—H10A	118.3
C3—C2—C1	117.85 (13)	C9—C11—C13	119.21 (16)
C3—C2—C6	120.60 (14)	C9—C11—H11A	120.4
C1—C2—C6	121.55 (13)	C13—C11—H11A	120.4
C4—C3—C2	120.01 (14)	C10—C12—C13	119.28 (16)
C4—C3—H3A	120.0	C10—C12—H12A	120.4
C2—C3—H3A	120.0	C13—C12—H12A	120.4
C5—C4—C3	117.84 (14)	C11—C13—C12	117.19 (14)
C5—C4—H4A	121.1	C11—C13—C14	121.72 (13)
C3—C4—H4A	121.1	C12—C13—C14	121.08 (14)
N1—C5—C4	124.29 (14)	C15—C14—C16	117.39 (14)
N1—C5—H5A	117.9	C15—C14—C13	121.31 (14)
C4—C5—H5A	117.9	C16—C14—C13	121.29 (13)
O2—C6—O1	122.82 (14)	C18—C15—C14	119.29 (15)
O2—C6—C2	122.94 (14)	C18—C15—H15A	120.4
O1—C6—C2	114.24 (13)	C14—C15—H15A	120.4
C8—C7—S1	117.93 (12)	C17—C16—C14	119.04 (15)
C8—C7—H7A	107.8	C17—C16—H16A	120.5
S1—C7—H7A	107.8	C14—C16—H16A	120.5
C8—C7—H7B	107.8	N3—C17—C16	123.77 (17)
S1—C7—H7B	107.8	N3—C17—H17A	118.1
H7A—C7—H7B	107.2	C16—C17—H17A	118.1
O4—C8—O3	124.16 (16)	N3—C18—C15	123.39 (15)
O4—C8—C7	122.06 (17)	N3—C18—H18A	118.3
O3—C8—C7	113.72 (14)	C15—C18—H18A	118.3
C10—N2—C9	117.01 (15)		
			
C5—N1—C1—C2	−0.4 (2)	C10—N2—C9—C11	−1.7 (3)
C5—N1—C1—S1	178.95 (11)	C9—N2—C10—C12	0.4 (3)
C7—S1—C1—N1	−0.26 (12)	N2—C9—C11—C13	1.7 (3)
C7—S1—C1—C2	179.13 (11)	N2—C10—C12—C13	0.7 (3)
N1—C1—C2—C3	0.8 (2)	C9—C11—C13—C12	−0.5 (2)
S1—C1—C2—C3	−178.52 (11)	C9—C11—C13—C14	−179.32 (15)
N1—C1—C2—C6	−179.22 (12)	C10—C12—C13—C11	−0.6 (2)
S1—C1—C2—C6	1.43 (18)	C10—C12—C13—C14	178.20 (15)
C1—C2—C3—C4	−0.7 (2)	C11—C13—C14—C15	−27.4 (2)
C6—C2—C3—C4	179.39 (14)	C12—C13—C14—C15	153.82 (17)
C2—C3—C4—C5	0.1 (2)	C11—C13—C14—C16	151.38 (17)
C1—N1—C5—C4	−0.2 (2)	C12—C13—C14—C16	−27.4 (2)
C3—C4—C5—N1	0.3 (2)	C16—C14—C15—C18	−1.3 (2)
C3—C2—C6—O2	173.56 (15)	C13—C14—C15—C18	177.51 (15)
C1—C2—C6—O2	−6.4 (2)	C15—C14—C16—C17	1.3 (2)
C3—C2—C6—O1	−6.0 (2)	C13—C14—C16—C17	−177.48 (15)
C1—C2—C6—O1	174.06 (14)	C18—N3—C17—C16	−0.6 (3)
C1—S1—C7—C8	70.16 (12)	C14—C16—C17—N3	−0.4 (3)
S1—C7—C8—O4	−152.53 (14)	C17—N3—C18—C15	0.6 (3)
S1—C7—C8—O3	30.22 (19)	C14—C15—C18—N3	0.3 (3)

### Hydrogen-bond geometry (Å, °)

**Table:**

*D*—H···*A*	*D*—H	H···*A*	*D*···*A*	*D*—H···*A*
O1—H1B···N2^i^	0.86 (2)	1.79 (2)	2.6564 (18)	178 (2)
O3—H3B···N3^ii^	0.86 (2)	1.82 (2)	2.6618 (18)	167 (2)
C4—H4A···O4^iii^	0.93	2.55	3.213 (2)	128
C15—H15A···O2^ii^	0.93	2.39	3.0664 (19)	130
C18—H18A···o2^ii^	0.93	2.70	3.232 (2)	117

Symmetry codes: (i) *x*+1, *y*+1, *z*; (ii) −*x*+1, *y*−1/2, −*z*+1/2; (iii) −*x*+1, −*y*, −*z*.
